# Metagenomic analysis reveals rapid development of soil biota on fresh volcanic ash

**DOI:** 10.1038/s41598-020-78413-z

**Published:** 2020-12-08

**Authors:** Hokyung Song, Dorsaf Kerfahi, Koichi Takahashi, Sophie L. Nixon, Binu M. Tripathi, Hyoki Kim, Ryunosuke Tateno, Jonathan Adams

**Affiliations:** 1 Department of Earth and Environmental Sciences, The University of Manchester, Manchester, UK; 2 School of Natural Sciences, Department of Biological Sciences, Keimyung University, Taegu, 42601 Republic of Korea; 3 Department of Biology, Faculty of Science, Shinshu University, Matsumoto, 390-8621 Japan; 4 Korea Polar Research Institute, Inchon, Republic of Korea; 5 Celemics Inc., 19F, Bldg. A, BYC High City, 131, Gasandigital 1-ro, Gwumcheon-gu, Seoul, 153-718 Korea; 6 Field Science Education and Research Center, Kyoto University, Kyoto, 606-8502 Japan; 7 School of Geography and Ocean Sciences, Nanjing University, Nanjing, 210023 Jiangsu China

**Keywords:** Biodiversity, Microbial ecology, Ecology, Ecology, Environmental sciences

## Abstract

Little is known of the earliest stages of soil biota development of volcanic ash, and how rapidly it can proceed. We investigated the potential for soil biota development during the first 3 years, using outdoor mesocosms of sterile, freshly fallen volcanic ash from the Sakurajima volcano, Japan. Mesocosms were positioned in a range of climates across Japan and compared over 3 years, against the developed soils of surrounding natural ecosystems. DNA was extracted from mesocosms and community composition assessed using 16S rRNA gene sequences. Metagenome sequences were obtained using shotgun metagenome sequencing. While at 12 months there was insufficient DNA for sequencing, by 24 months and 36 months, the ash-soil metagenomes already showed a similar diversity of functional genes to the developed soils, with a similar range of functions. In a surprising contrast with our hypotheses, we found that the developing ash-soil community already showed a similar gene function diversity, phylum diversity and overall relative abundances of kingdoms of life when compared to developed forest soils. The ash mesocosms also did not show any increased relative abundance of genes associated with autotrophy (*rbc, coxL*), nor increased relative abundance of genes that are associated with acquisition of nutrients from abiotic sources (*nifH*). Although gene identities and taxonomic affinities in the developing ash-soils are to some extent distinct from the natural vegetation soils, it is surprising that so many of the key components of a soil community develop already by the 24-month stage. In this system, however, rapid development may be facilitated by the relatively moderate pH of the Sakurajima ash, proximity of our mesocosms to propagule sources, and the rapid establishment of a productive bryophyte and lichen layer on the surface. Ash from other volcanoes richer in acids or more distant from propagule sources could show a different pattern and slower soil biota development.

## Introduction

Volcanic ash is one of the world’s main sources of soils, particularly in the more active regions of the Pacific ‘ring of fire’, such as Japan^1,2^. Unlike its namesake wood ash, volcanic ‘ash’ is a powder of silicate minerals, and when it first falls it is sterile or nearly so, and essentially free of soluble nutrients^3^. The ash also has little water retaining capacity and erodes easily. Chemical and biological processes are required to release nutrients, and contribute organic matter to sustain food webs^3^. It is clear that on a timescale of decades, volcanic ash can become a fertile soil with a diverse biota—and indeed the soils of much of Japan are developed over volcanic ash^1,2^. The eventual result of most successions on volcanic ash in Japan and elsewhere are organic rich soils under either forest or (in colder environments) scrub and tundra^2^.

Studies on developing volcanic ash soils have so far concentrated on chemical characterisation of the ash soils^4–7^ or description of bacterial communities using only 16S rRNA gene sequencing^8–11^. There have been few studies of the functional aspects of the biota of developing ash soils. In one exception, a metagenome study by Fujimura et al.^3^ compared the functional gene profile of 3.5-, 6.6-, and 9.5-year old ash from the Miyakejima volcano in Japan and noted that the *rbcL* gene for CO_2_ fixation and *nifH* gene for nitrogen fixation were more abundant in the developing ash-soils. These genes have been suggested to be important for energy, carbon and nutrient acquisition of microbes in ash soils that lack organic matter^12–16^. However, the study of Fujimura et al.^3^ did not consider the even earlier stages—the first 2 years—of biota development. The ash system they studied was also one that was relatively acidic at inception, being rich in sulphuric acid, while many other ash deposits are not so acidified^3^.

The relative paucity of detailed studies on ecosystem development on volcanic ash may be partly explained by the fact that volcanic explosion events are sporadic and unpredictable, and the resulting ash field sites are often off-limits for sampling for several years afterwards due to safety concerns. While observations of macroscopic life in the earliest stages of volcanic ash succession have long been recorded^17–19^, there have been few or no studies of soil biota during the first 2 or 3 years of soil biota development in volcanic ash. Most studies of soil biota have begun at five or more years after the ash initially fell^20,21^, partly because most areas of ash deposition are off limits for sampling for several years until volcanic activity has subsided. Here we set out to circumvent some of the practical difficulties of observing soil development processes on freshly fallen volcanic ash by setting up outdoor mesocosms positioned in various environments across Japan, using the same source of freshly fallen volcanic ash from the Sakurajima volcano, and by using a metagenome approach. Our intention was to understand how rapidly a soil biota appears in the ash, how it may differ from more developed soils, and whether climate and location have a large effect on the development of biota.

The major questions behind this study were:

*1) How rapidly can the major functional components of a soil ecosystem appear in volcanic ash?* We hypothesized that due to the relatively extreme conditions in the ash—such as lack of available mineral nutrients and organic matter—and also due to limitations on arrival and establishment of biota due to slow dispersal, there would be: (1) a lower diversity of broad taxonomic categories of organisms, such as phyla of fungi, archaea, Protista and Metazoa than are normally found in a developed soil. (2) A lower diversity of categories of functional genes of soil biota than are normally found in a developed soil, indicating less functional complexity in the early ash-soil system.

*2) Which distinct functional aspects characterize the early development of ash-soil biota?* We hypothesized that the distinct chemical environment of the developing ash would result in increased relative abundance of a number of gene categories. For example: (1) Increased relative abundance of genes associated with autotrophy (e.g. *rbc* gene and *coxL* gene) due to lack of organic matter^12–16^, and abundance of photosynthetic and chemoautotrophic groups of microbes^8,21–23^. Since the usual heterotrophic routes are assumed not to be available initially in the newly developing ash ecosystem, and oxidizable minerals are abundant, microbial chemosynthesis may be expected to be relatively more important than in a developed soil. (2) Increased relative abundance of genes that are associated with acquisition of nutrients from abiotic sources rather than decomposition of organic matter (e.g. *nif* genes). (3) Increased relative abundance of stress response genes and dormancy related genes, due to more frequent drying of the ash–soil which will presumably be well drained and low in organic matter. (4) Decreased relative abundance of interaction related genes (CRISPR genes and genes classified as regulation and cell signalling and virulence genes in the SEED subsystem database^24^).

## Results

### Soil physicochemical conditions

Supplementary Fig. S1 shows total carbon and total nitrogen concentrations and pH of the ash soil and forest soils at each site. Total carbon and total nitrogen concentrations were significantly lower in the ash soils, irrespective of location and age. pH was also significantly lower in the ash soils in Sakurajima and Takakuma, but no such pattern was found in other sites. At Norikura Low, the third year ash soil had higher pH.

### Bacterial community composition and diversity analysed based on the 16S rRNA gene amplicon sequencing

**Figure 1:**
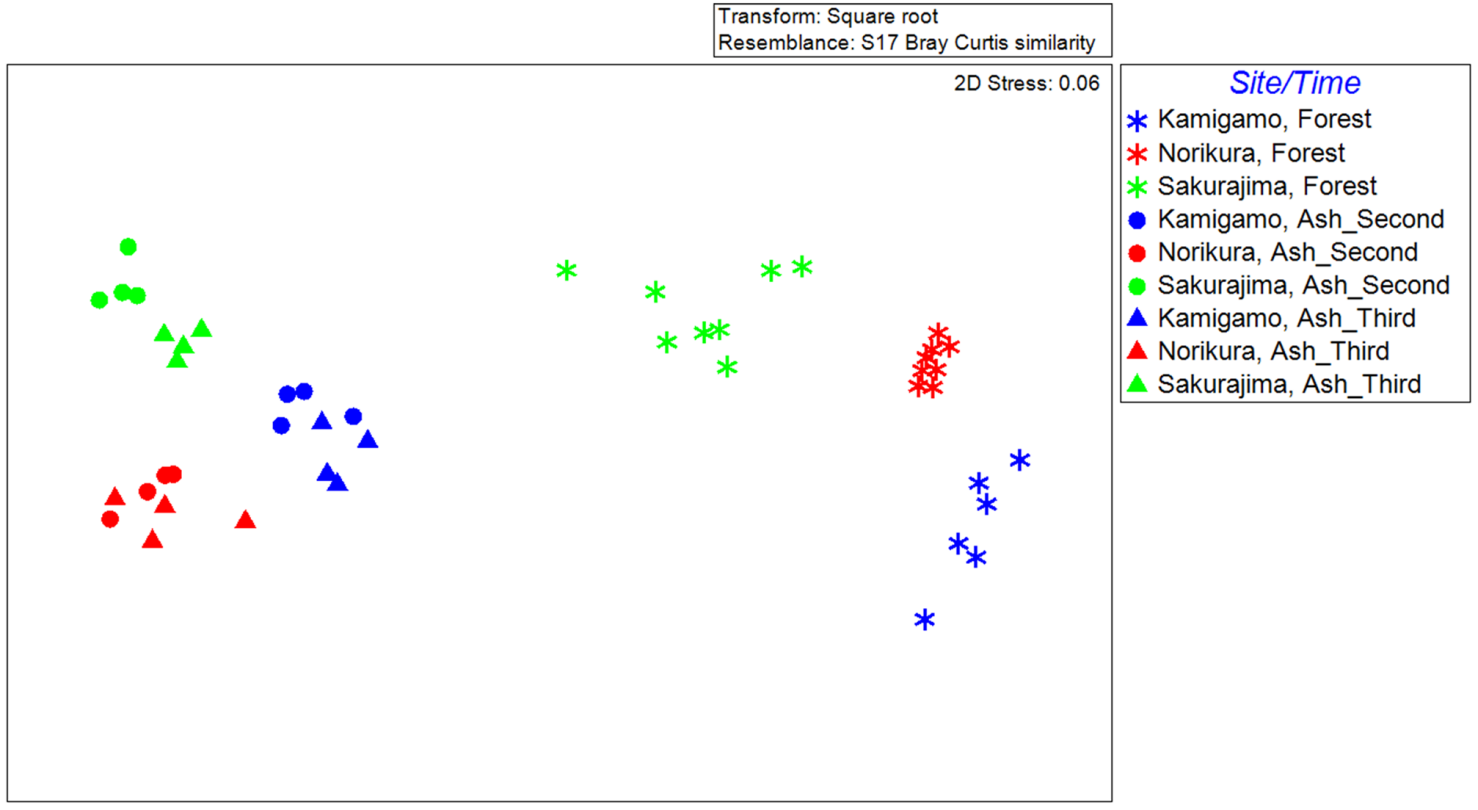
nMDS plot drawn based on Bray–Curtis dissimilarity of bacterial community between samples, based on amplicon sequencing which new 36 month results added. Second = 24 month, third = 36 month.

Two way crossed ANOSIM test results showed significant differences in bacterial community composition between 2nd year ash pots, 3rd year ash plots and natural forest soils (Fig. 1, Supplementary Table S1). Also, the community composition was significantly different between sites (Fig. 1, Supplementary Table S1). The Bray–Curtis distances of the 2nd-year pot samples and the forest samples were not significantly different from the Bray–Curtis distances of the 3rd-year pot samples and the forest samples at Norikura and Sakurajima. However, the Bray–Curtis distance between the 2nd-year ash soils and forests was significantly higher than the Bray–Curtis distance between the 3rd-year ash soils and forests at Kamigamo (Supplementary Fig. S2).

For both 24 and 36 month samples, we found significant differences between ash-soils and natural forest soils in the relative abundance of major bacterial phyla (Supplementary Fig. S3), continuing the trend established for the 24 month samples by Kerfahi et al.^10^. The relative abundance of Acidobacteria was significantly lower (p < 0.05) in the ash soils compared to forest at all sites except for Sakurajima, where the 3rd-year ash soil showed no significant difference in the relative abundance of Acidobacteria compared to forest soil (p = 0.459). The relative abundance of Patescibacteria was also overall lower in the ash soils compared to forest (no significant difference was found at Kamigamo, but the trend was similar). In contrast, the relative abundance of Chloroflexi in the ash soils was significantly higher compared to the forest soils at all sites.

Among the major bacterial families (Supplementary Fig. S4), we found significantly higher relative abundance of Ktedonobacteraceae in the ash soils compared to forest soils at all sites. The relative abundance of Acetobacteraceae was also higher in the ash soils in all sites except for Sakurajima, where there was no significant difference in the relative abundance of Acetobacteraceae found between the 3rd-year ash soils in comparison with forest (p = 0.349). In contrast, the relative abundance of Xanthobacteraceae was significantly higher in the forest soils at Norikura and Sakurajima but no significant difference found at Kamigamo. The relative abundance of Pedosphaeraceae was always higher in the forest soils regardless of location and age.

**Figure 2:**
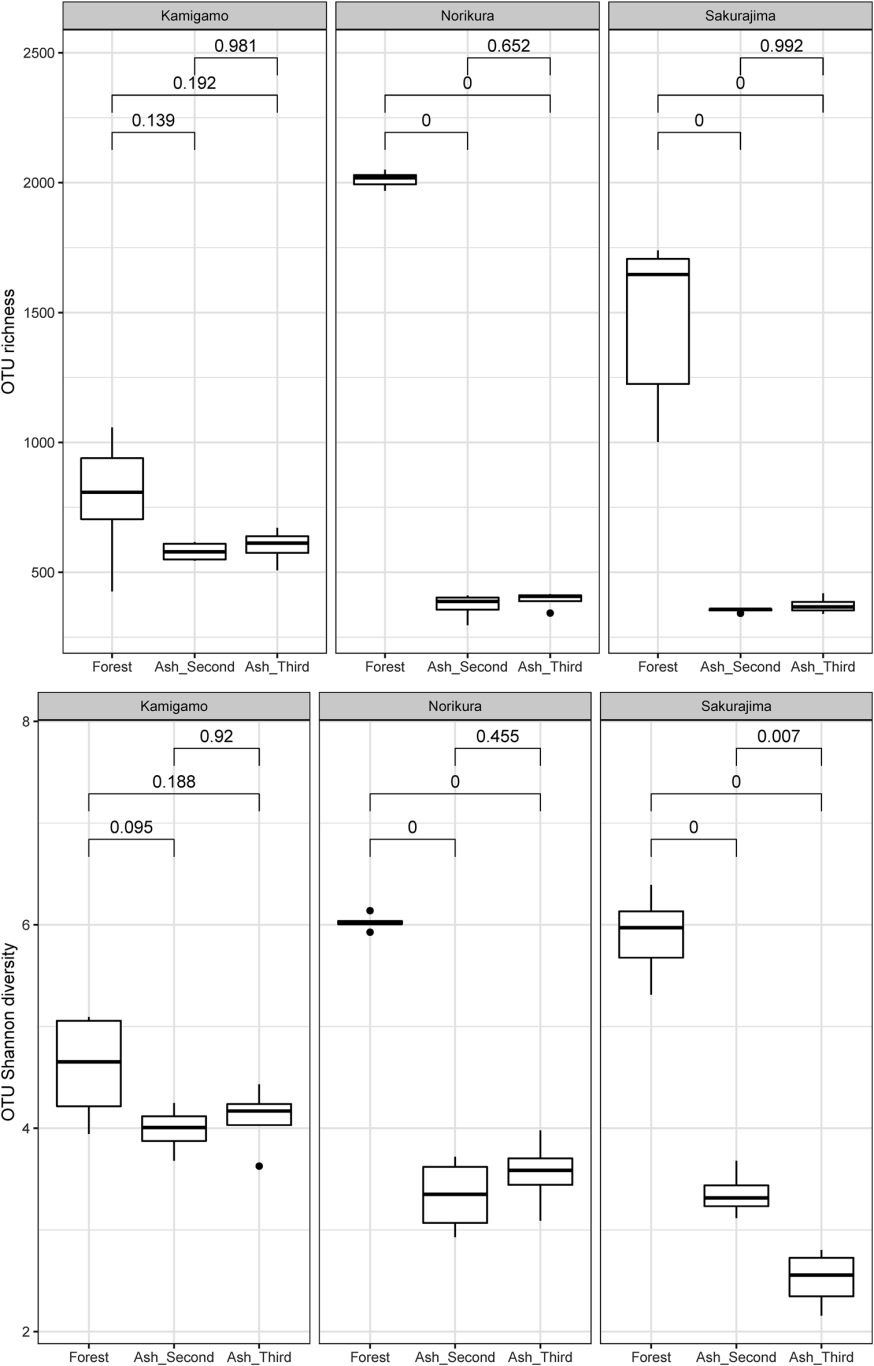
Box plots showing OTU richness (upper) and OTU Shannon diversity based on 16S rRNA amplicon sequencing.

The total richness of bacterial OTUs and Shannon diversity of the forest soils were significantly higher than in the ash soils at Norikura and Sakurajima, for both 24 months and for 36 months (Fig. 2). There was no significant difference found at Kamigamo, but the trend was similar.

### Comparison of bacterial community composition and diversity between 16S rRNA and metagenome sequencing

Family level bacterial community composition based on the metagenome sequencing data are shown in Supplementary Fig. S5. The major families identified by metagenome sequencing were similar to those identified by 16S rRNA amplicon sequencing (Supplementary Fig. S4). However, the relative abundance of minor families in the metagenomes was higher than identified by 16S rRNA amplicon sequencing. Also, the proportion of unclassified/unidentified sequences was lower in the metagenome sequencing data. Family diversity accumulation curve shows higher diversity of families calculated based on metagenome sequencing data (Supplementary Fig. S6).

### Phylogenetic assembly and turnover of bacterial community

SES.MNTD values calculated based on 16S rRNA data in all samples were below zero suggesting that co-occuring OTUs were phylogentically more related than expected by chance (Supplementary Fig. S7). SES.MNTD values of the natural forest soils were significantly lower than the ash soils suggesting stronger phylogenetic clustering in the forest soils at Norikura and at Sakurajima (Supplementary Fig. S7). However, no significant difference was found at Kamigamo.

### Archaeal and eukaryotic phyla

In total, five archaeal phyla were found in the metagenomes in this study (Crenarchaeota, Euryarchaeota, Korarchaeota, Nanoarchaeota, Thaumarchaeota). The number of archaeal phyla annotated based on metagenome sequence data was quite consistent, varying only from four to five phyla amongst the various samples. The number of eukaryotic phyla annotated based on metagenome sequence was significantly different between ash soils and forest soils in Kamigamo and Takakuma (Supplementary Fig. S8). In both sites, forest soil had a lower total number of Eukaryotic phyla. The phyla that were present in the ash-soil samples but not in any forest samples were all minor taxonomic groups which had relative abundance of 0–0.032%. The overall non-bacterial phylum composition was similar between the ash and the forest samples (Supplementary Fig. S9).

### Bacterial functional gene composition analyzed based on metagenome sequencing

As most of the sequences annotated based on the metagenome sequencing were bacterial (on average, 97% of reads were bacterial)—and in order to make comparison with the findings based on the 16S rRNA gene amplicon sequencing—we have only focused on bacterial functional genes in this and the following section.

**Figure 3:**
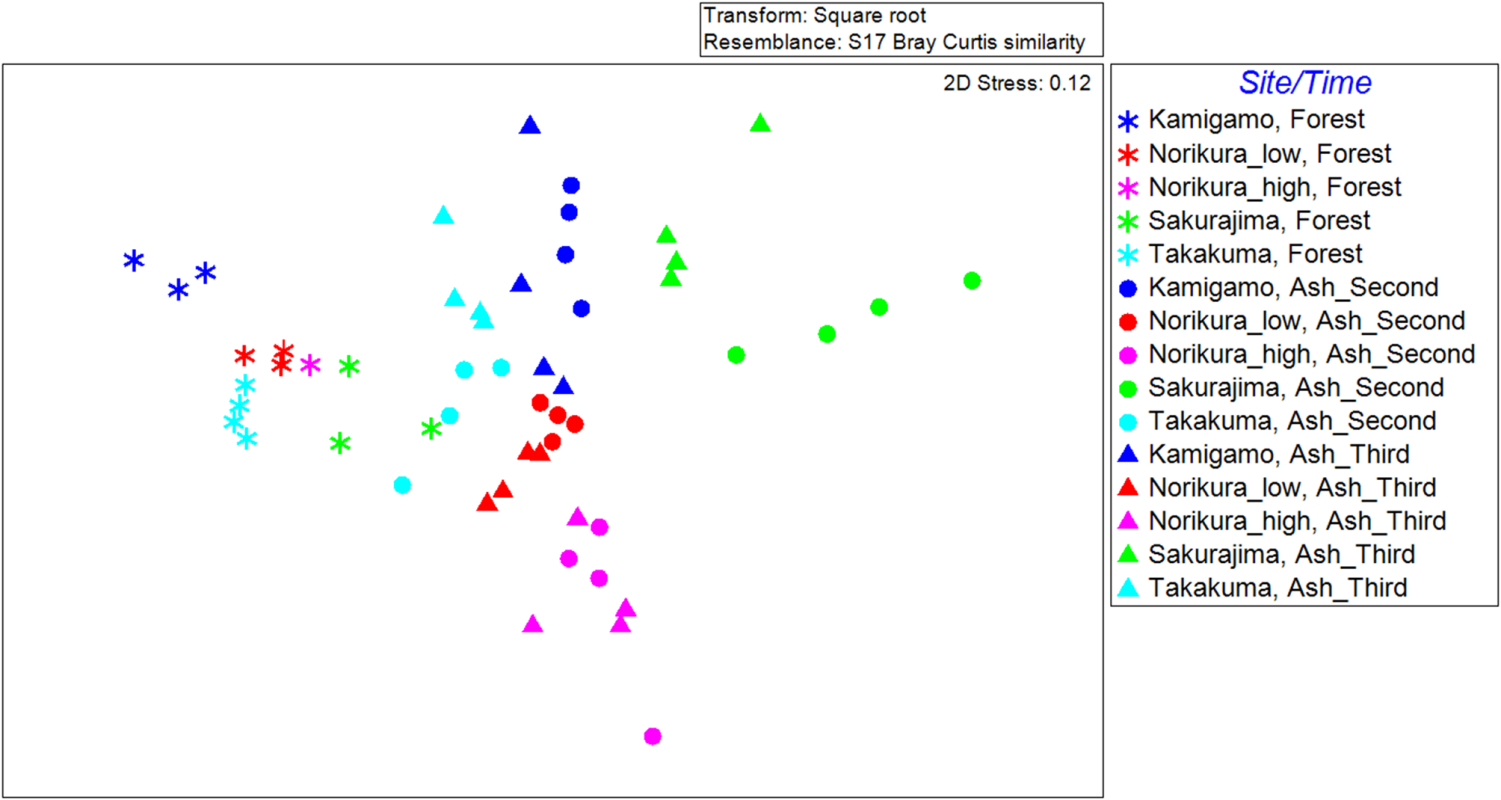
nMDS plot drawn based on Bray–Curtis dissimilarity of Subsystem level4 functional gene composition between samples.

**Figure 4:**
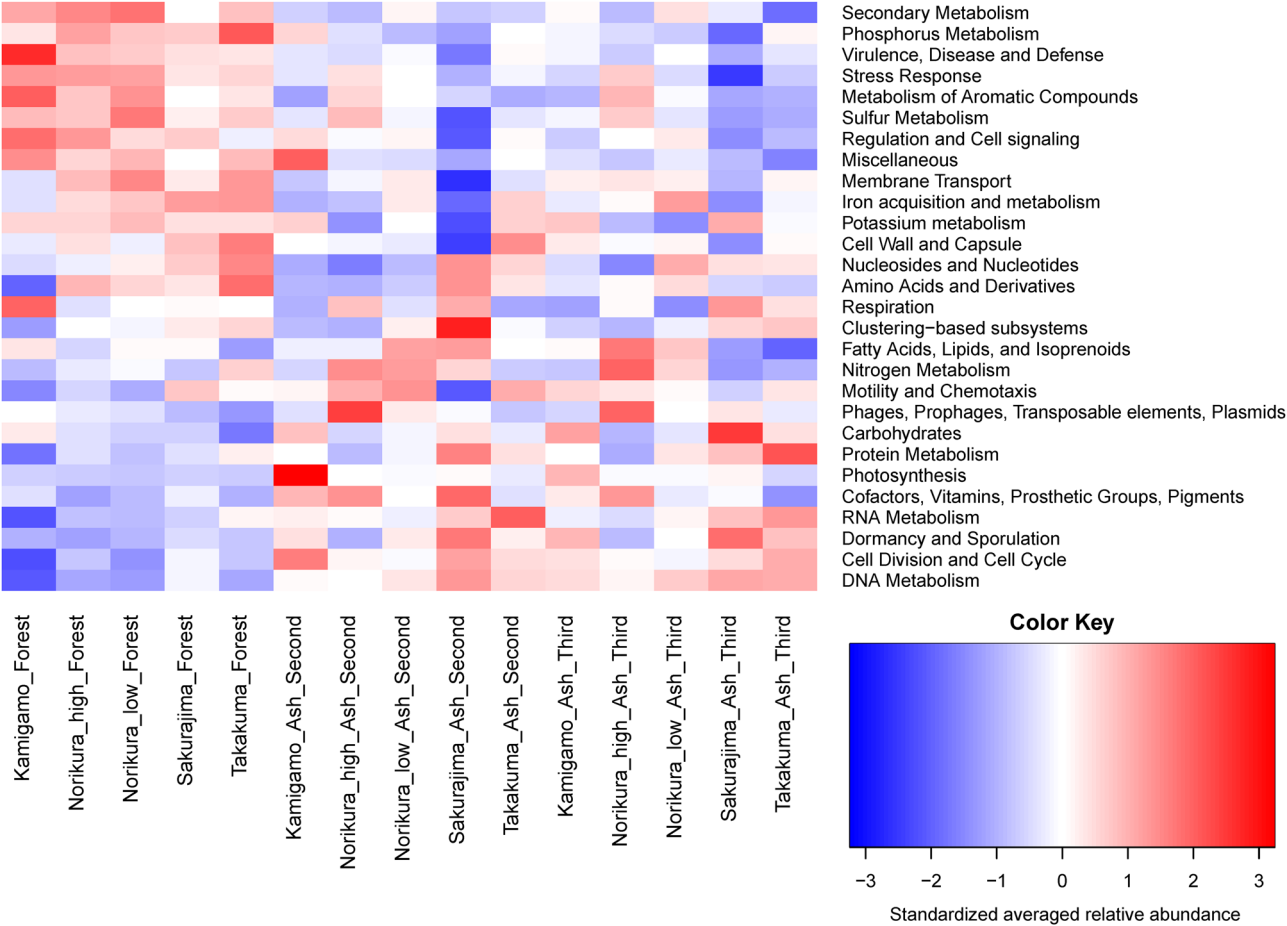
Heatmap of standardized averaged relative abundance of Level 1 functional gene categories.

Two way crossed ANOSIM test results showed a significant difference between the 2nd-year ash soils, 3rd-year ash soils, and forest soils in terms of bacterial functional gene composition (Fig. 3, Supplementary Table S2). Also, the community composition was significantly different between sites (Fig. 3, Supplementary Table S2). The Bray–Curtis distances of the 2^nd^-year pot samples and the forest samples were not significantly different from the Bray–Curtis distances of the 3^rd^-year pot samples and the forest samples at all sites (Fig. S10). Figure 4 shows the heatmap of standardized averaged relative abundance of Level 1 functional gene categories.

**Figure 5:**
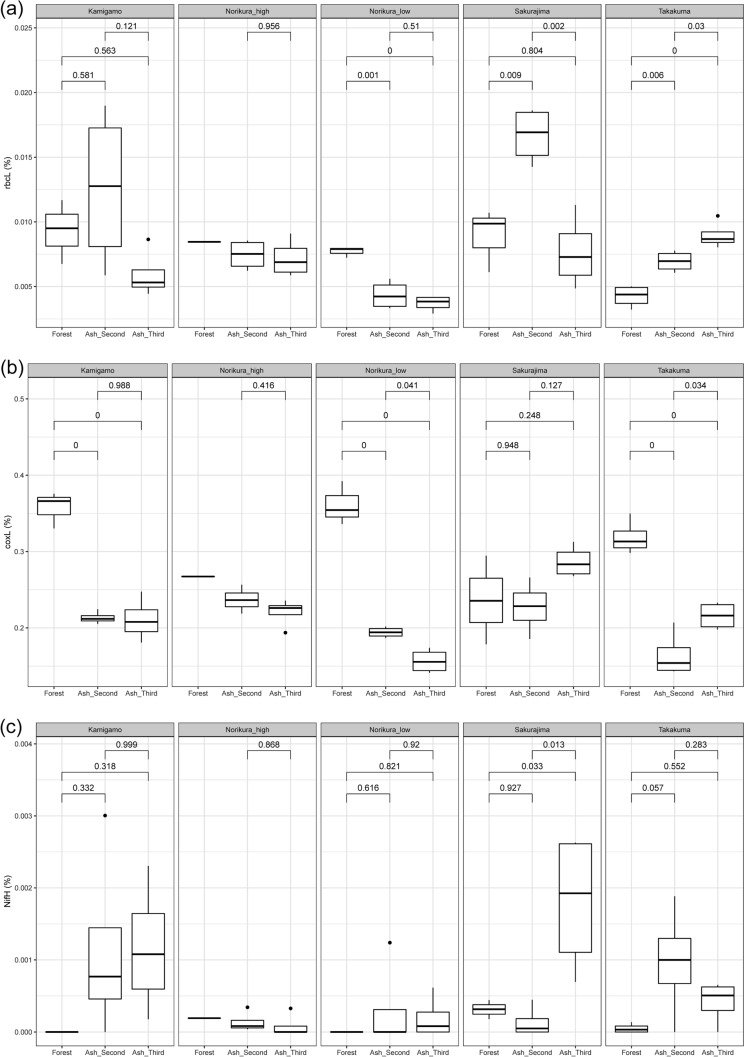
(**a**) Relative abundance of *rbcL* (ribulose bisphosphate carboxylase large chain) gene in the ash soils and forest soils. (**b**) Relative abundance of *coxL* (Carbon monoxide dehydrogenase large chain) gene in the ash soil and forest soil. (**c**) Relative abundance of *nifH* gene in the ash soil and forest soil.

In terms of specific gene categories, we found the relative abundance of *rbcL* genes showed no consistent pattern (Fig. 5a). The relative abundance of these genes was highest in the forest at the lower elevation of Norikura compared to the ash mesocosms, but the opposite pattern was shown in Takakuma. In contrast to our expectation, the relative abundance of *coxL* genes was higher in the forest soils (Fig. 5b) in most of the sites except for Sakurajima. The relative abundance of *nif*H genes was very low overall, and showed no significant difference between forest and ash soils (Fig. 5c).

**Figure 6:**
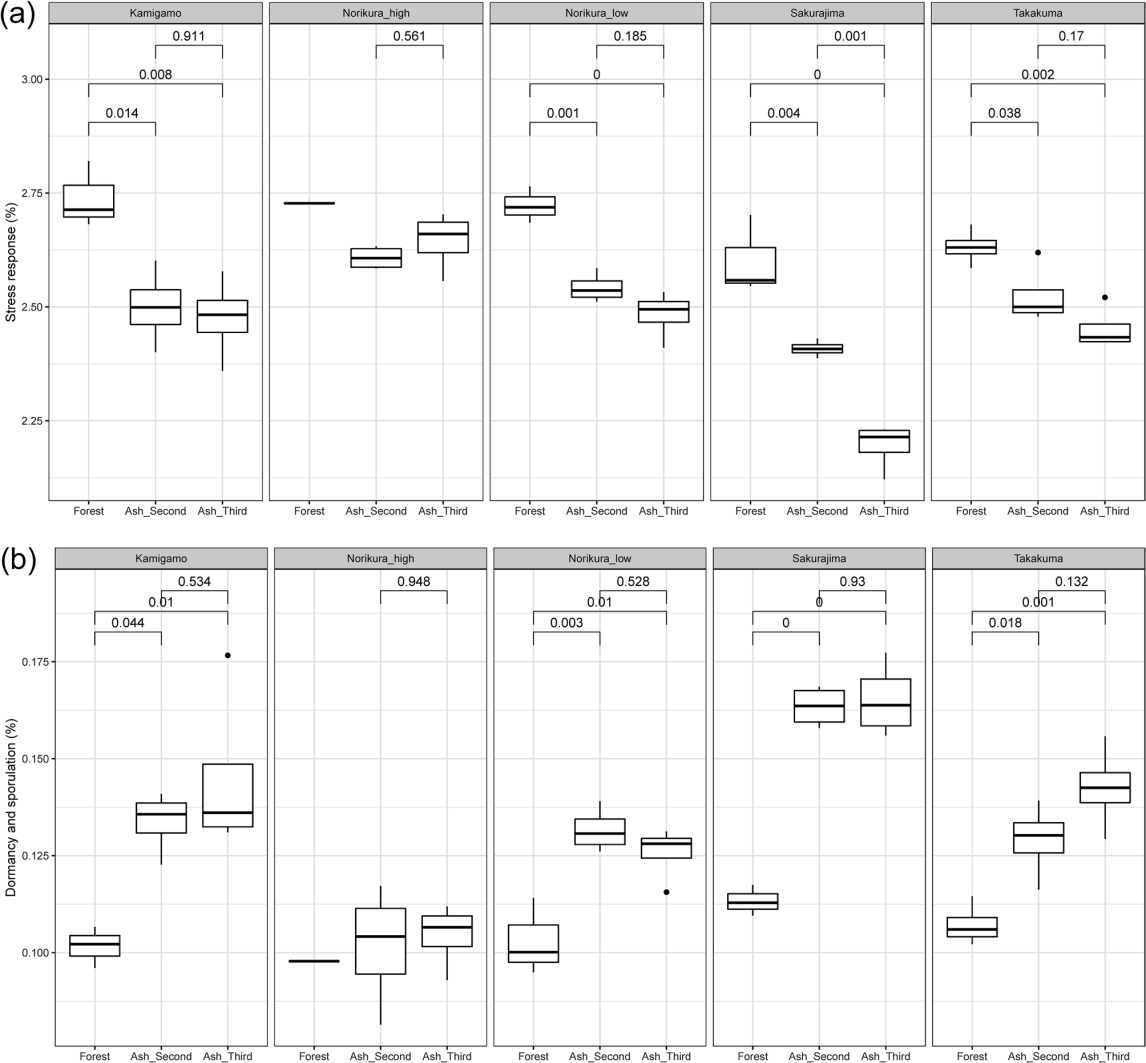
(**a**) Relative abundance of stress response gene category in the ash soil and forest soil. (**b**) Relative abundance of dormancy and sporulation gene category in the ash soil and forest soil.

**Figure 7:**
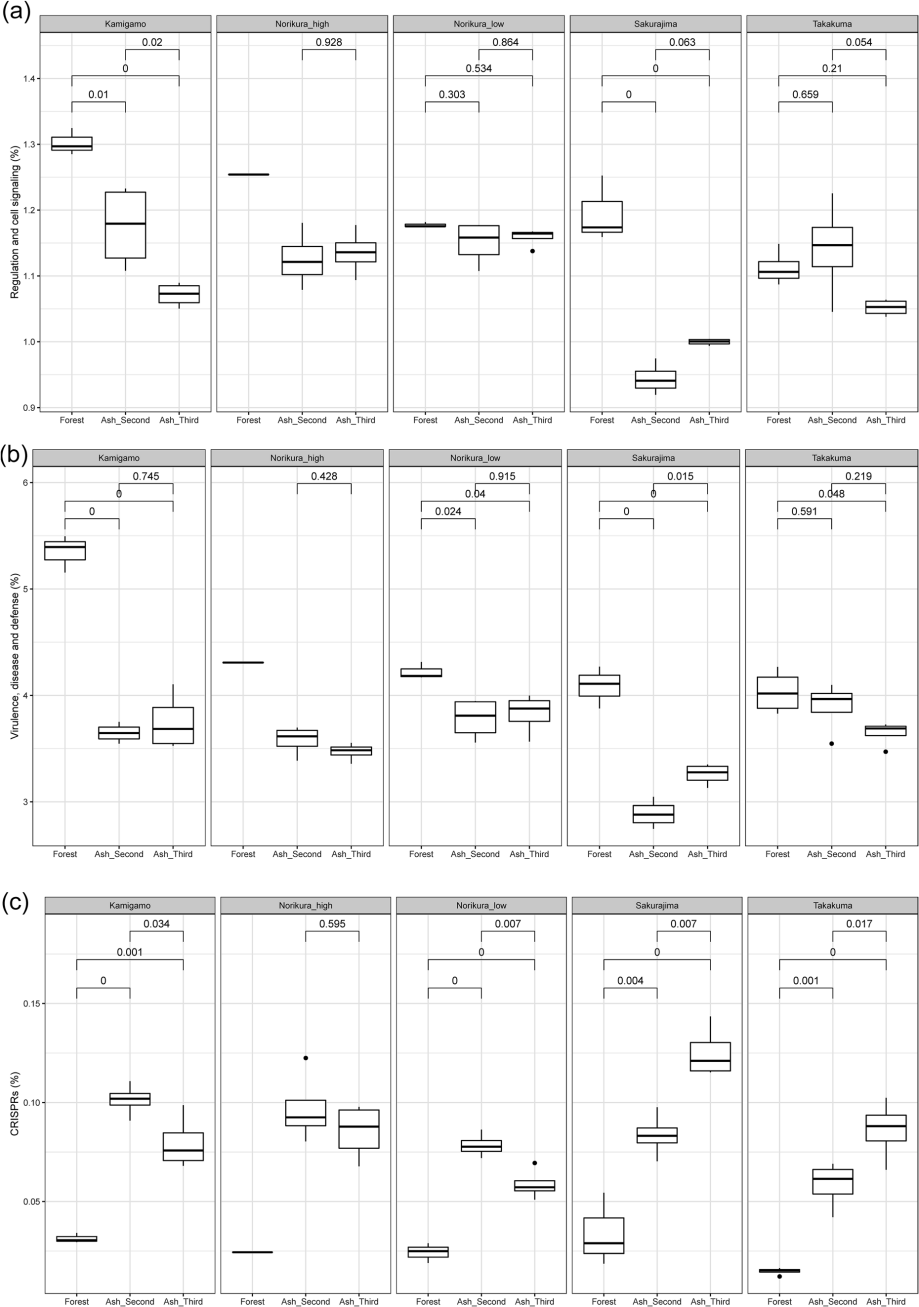
(**a**) Relative abundance of regulation and cell signalling gene category in the ash soil and forest soil. (**b**) Relative abundance of virulence, disease, and defense gene category in the ash soil and forest soil. (**c**) Relative abundance of CRISPR genes in the ash soil and forest soil.

The relative abundance of stress response genes was in general, higher in the forest soils (Fig. 6a). Dormancy and sporulation related genes were relatively more abundant in the ash soils (Fig. 6b). Gene categories related to cell–cell interactions were relatively more abundant in the forests in most of the sites (Fig. 7a,b). However, the relative abundance of CRISPR genes was lower overall in the forest soils (Fig. 7c).

### Bacterial functional gene diversity

**Figure 8:**
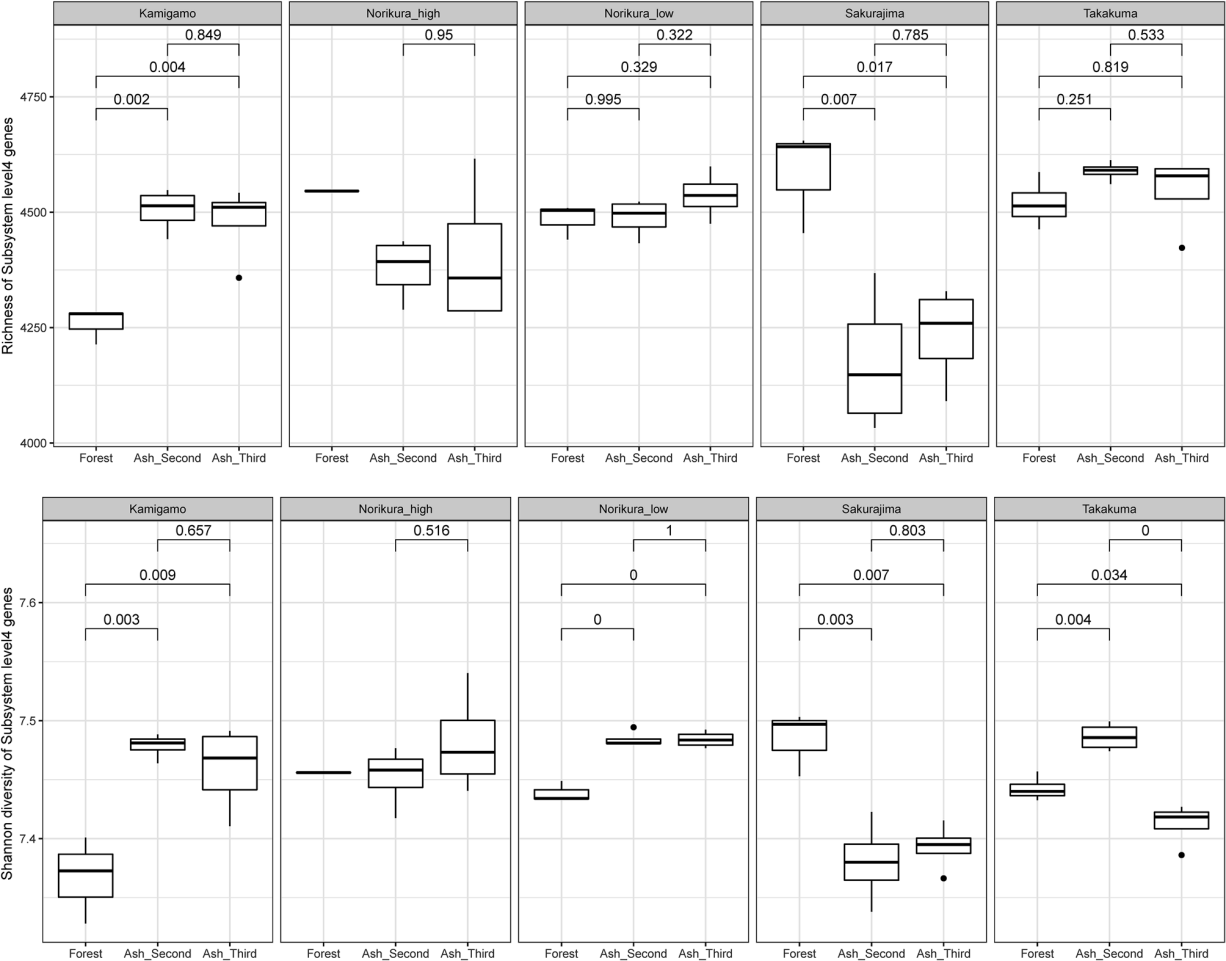
Box plots showing richness of Subsystem level 4 functional genes (upper) and Shannon diversity of Subsystem level 4 functional genes (lower).

Figure 8 shows that there were no consistant trends found in the functional gene diversity between ash soils and forest soils. Functional gene diversity was higher in forest soils than in ash soils at Sakurajima. However, the opposite pattern was found at Kamigamo. There was no significant difference in the functional gene diversity between ash soils and forest soils in Norkura and Takakuma.

## Discussion

### How rapidly can the major functional components of a soil ecosystem appear in volcanic ash?

The overall conclusion from comparing the functional composition of the developing ash-soil with natural forest soils from the same climates, is that the major biological aspects of the system have developed surprisingly quickly. Already by the 24-month and 36-month stage, the ash-soil had a similar diversity of bacterial functional genes to a forest soil (Fig. 8).

This contrasts with the findings of Fujimura et al.^3^, who found that ash deposited by the Miyakejima volcano still showed a very distinct and low diversity community compared to that expected of normal forest soils. This difference could be attributable to differences in the initial chemical composition of the ash: the pH value of the Miyakejima ash studied by Fujimura et al.^3^ was much lower than the ash we sampled, due to the persistence of acids from the initial eruption, or acidic rainfall from later outgassing. Our Sakurajima ash samples had only a slightly acidic pH (5.2) at the time of harvesting, which was similar to the average value (5.14) of 251 ash samples from Sakarajima collected from 1955 to 2001^25^, and they remained in the 5–6 pH range at both 24 months and 36 months. Even the ash soil mesocosm samples that were situated on the lower slopes of the Sakurajima volcano, and the nearby natural forest soils at Sakurajima, remained in the pH 5–6 range and had a similar gene composition to other sites, suggesting that the area around the Sakurajima volcano is not subject to frequent highly acidic rain events. The difference between the Sakurajima ash and the Miyakejima ash hints at the importance of details of the chemical environment of the ash for determining its development into a functioning soil ecosystem.

Another factor which may explain the difference between the paths of biota development of the ash from the two volcanoes is the relative availability of propagules. Our ash soils were surrounded by developed ecosystems which could provide a rain of propagules of microorganisms and bryophytes in windblown dust or in rain splash. By contrast the Miyakejima ash field was an area of extensive ecosystem destruction. If bryophytes and lichens had not been able to establish due to unavailability of propagules, an important source of photosynthetically-fixed energy would have been unavailable to the Miyakejima system. This would have impeded the development of a decomposer food chain, of mineral weathering by organic acids and chelates, and of buffering of soil pH and water content and nutrient storage on organic matter and clay surfaces.

### Was there a lower taxonomic diversity than is normally found in a developed soil?

At the broadest level, from the metagenomes, the taxonomic composition of the biota of the developing ash-soil resembled that of the developed forest soils—with archaea, fungi, protists and metazoans occurring at similar relative abundances in the ash-soils and forest soils. In this sense, the ash-soil had developed remarkably quickly in terms of the basic taxonomic framework of a functioning soil ecosystem. Bryophytes and lichens were able to establish^10,26^ with their propagules small enough to enter through the gauze covering, and presumably played an important role in providing carbon and extra niches to the developing ecosystem—even though they are unlikely to be as supportive of a diverse soil biota as the extensive root systems of vascular plants.

There was however—continuing a pattern seen in the 24-month results reported by Kerfahi et al.^10^—a lower OTU and Shannon diversity of bacteria based on the 16S rRNA amplicon sequencing. The continuing lower OTU-level 16S taxonomic diversity of the ash soils might have had multiple causes.

Firstly, limitations on dispersal and colonization of the ash soil systems might have kept OTU diversity lower. It is assumed that the biota present were mostly derived from the surrounding forest ecosystems—as windblown or rain splashed material. Dispersal limitation of soil biota is likely to play some role in all volcanic ash fields, although the existence of nearby areas of surviving ecosystem is also common^20,27^. Most volcanic explosions or ash fields result in no more than a few square kilometres of landscape being completely devastated, and isolated pockets of vegetation that survive are common^27,28^—in this respect the proximity of our mesocosms to natural vegetation may be fairly realistic. In natural ash deposits, upwards colonization by soil biota from buried ‘legacy soils’ is also possible^20^. In our experiment, upwards colonization from soil below the pots was not possible, and the plastic gauze covering may have prevented access by insects, birds or seeds that could have brought incidental soil biota. It is thus difficult to know for sure whether the overall role of dispersal limitation in our mesocosm systems is stronger or weaker than would generally be the case in natural ash fields. Since natural ash fields themselves are also very heterogenous in terms of area, depth and degree of devastation of natural vegetation, it is especially hard to generalise.

Secondly, the relatively extreme chemical and physical conditions of the volcanic ash itself are also likely play a major role in limiting the bacterial taxonomic diversity of the ash-soil biota in the mesocosms. Although the pH of the ash in our mesocosm systems was not extreme, it may be expected to be droughty due to low organic matter content, and low in available nutrients and organic matter that could sustain soil food chains. Even by 36 months, the organic carbon of the ash-soil was still orders of magnitude lower than in the surrounding forest soils (Supplementary Fig. S1)^10,26^. Thus, fewer niche types may be viable in the environment of the developing ash.

Nevertheless, although lower level taxonomic diversity of bacteria is clearly lower than in the established forest soils, the metagenome results from the ash soil mesocosms show that most higher level taxonomic groups of bacteria, archaea and eukaryotes are already present by the 24-month and 36-month stage, emphasizing that in some respects the establishment of soil biota has been rapid. While DNA of dead microorganisms blown as dust could have provided the impression of populations being present, it seems unlikely that dead material would be able to disperse in the same relative proportions as in a forest soil, to give a metagenome that resembled a developed soil in all its major groups, in roughly the same relative abundances (Please refer to Fig. 1 in Kerfahi et al.^10^).

### Was there a lower diversity of categories of functional genes of soil biota than are normally found in a developed soil, indicating less functional complexity in the early ash-soil system?

Surprisingly, the richness and diversity of functional gene categories at Level 4 of the SEED Subsystem was not significantly different between the ash mesocosms and the forest soils. Around 97% of the metagenome reads in the ash mesocosms and the forest soil were bacterial, so the much lower OTU diversity of bacteria in the mesocosms would be expected to give a lower functional diversity of genes. The contrast between patterns of taxonomic and functional diversity in the ash mesocosms emphasises the redundancy of gene functions in soil organisms—such that losing a high proportion of taxonomic diversity apparently has no effect on functional diversity. If the level of functional gene diversity is high, this implies that in a general sense the ecosystem can potentially perform a wide variety of functions. Greater functional diversity is also considered to result in greater resilience of the ecosystem^29^.

We had also hypothesized that the distinct chemical environment of the developing ash would result in increased relative abundance of a number of specific gene categories:

### Is there an increased relative abundance of genes (or of prokaryotic genera) associated with autotrophy (e.g.* Rubisco gene, coxL gene*) in the ash-soils?

We searched for potential taxa and genes which might indicate autotrophic carbon fixation through chemosynthetic oxidation processes. We found a higher relative abundance of Ktedonobacteraceae (Chloroflexi) in the ash soils compared to forest soils, which is a pattern that was also found in previous ash studies^21,30^. Ktedonobacteraceae is a novel taxonomic group that has only been recently added to the phylogenetic tree of Chloroflexi^30,31^. Their genome has been reported to be large and they are known to have a wide potential for different metabolic mechanisms^32^. Ktedonobacteraceae are mostly found in extreme environments, including volcanic ash and hydrothermal vents^30^.

We had hypothesized that the *coxL* (carbon monoxide dehydrogenase large chain) gene—which is implicated in autotrophy—would be more abundant in the ash soil samples. Likewise, the *rbcL* (ribulose bisphosphate carboxylase large chain) gene is involved in carbon fixation and we hypothesized its greater abundance in the ash-soil mesocosms. However, in fact both *rbcL* and *coxL* had higher relative abundances in the forest soils.

Although *coxL* has been mostly linked with chemotrophic organisms living in an extreme environment, due to its potential role for assisting heterotrophic growth in the environments lacking organic matter^21,33^, it is also one of the more abundant genes in natural forest soils^34^. Noting that natural forest is one of the largest global sinks of atmospheric CO^34,35^, the high relative abundance of *coxL* gene might be due to high abundance of CO in natural forests. Also, the coxL gene might have the potential to participate in other metabolic pathways. The presence of coxL genes in many different types of organisms (e.g. plant symbionts, animal pathogens, etc.) suggests other roles of CO-oxidation by the *coxL* gene^33^.

The higher abundance of *rbcL* in the forest soils in our study might be related to photosynthetic C fixation by cyanobacteria in both the forest soil and ash soils. *rbcL* gene also have been found in many natural forest sites. By contrast, Fujimura et al.^3^ found *rbcL* to be more abundant in developing volcanic ash soils, which might be attributable to differences in the chemical composition of ash or due to details of the mesoocosm system used in our study.

### Is there increased relative abundance of genes that are associated with acquisition of nutrients from abiotic sources rather than decomposition of organic matter (e.g. nitrogen fixation genes)?

Contrary to expectations we found no differences in the relative abundance of nitrogen fixation gene (*nifH*) between the ash soils and forest soils. It is possible that this reflects nitrogen being relatively more limiting in the forest soils, where there is an abundance of organic carbon. It is also possible that in the forest soils, the abundance of labile carbon in the rhizosphere of N-limited plants encourages N fixing bacteria through root secretions^36^.

### Is there increased relative abundance of stress response genes and dormancy related genes?

As hypothesized, we found that dormancy related genes were more abundant in the ash mesocosms than in the forest soils. This would seem to be adaptive in terms of survival of a well-drained ash-soil, low in organic matter, which is apt to dry out. This would depend, however, on the detailed microclimate. Field measurements suggested that in sunny conditions the microclimate in the static air in the trays in which our pots were held was generally around 0.5–1 °C higher than in the open air a meter away, although this difference disappeared in cloudy conditions and at night^10^. It is possible that our mesocosms (exposed in open sunlight, but covered by a gauze) would have either retained or lost moisture more easily than the soils, and that this might have affected the frequency of drying. We had also anticipated that stress response genes would be more common in the ash mesocosms. However, in this case the stress response category was less common than in the forest soil, surprisingly suggesting that at a cellular level stresses are in fact less common in the ash-soil.

### Is there decreased relative abundance of cell–cell interaction related genes?

As anticipated, genes related to cell–cell interactions such as regulation and cell signalling genes and virulence, disease, and defense genes (Fig. 7a,b) were relatively less abundant in the ash mesocosms. This seems to emphasize that complex interactions are less common in the developing ash system than in a fully developed soil with its more abundant and diverse carbon sources. However, bacterial genes found as part of CRISPRs were relatively less abundant in the forests, implying lower intensity of defense mechanisms of bacteria against viruses in the forests compared to ash soils. The relatively low OTU diversity of the ash soils may perhaps allow greater spread and mortality of viruses through populations, necessitating greater investment in defences.

Since Odum^37^, it has been noted that developing ecosystems tend to be based on tighter and more effective interactions as succession continues. For example, Morriën et al.^38^ found that during secondary succession of old field ecosystems, co-occurrences of taxa become more predictable and carbon cycling in the decomposer chain more efficient. Empirically our findings seem to agree with the same paradigm, as we found cell–cell interaction genes found to be more abundant in the developed forest soils. It would be very interesting to directly measure decomposer carbon cycling efficiency in ash-soil mesocosm systems as they develop.

Although we focused on 16S rRNA amplicon sequence data for comparing OTU level diversity between samples as taxonomic assignment based on MG-RAST pipeline is very limited^39,40^, we had briefly compared the family level bacterial community composition assigned based on metagenome sequence data and based on 16S rRNA amplicon sequence data. It is still debatable if whole genome sequencing, which is free from primer bias, is superior in this respect to 16S rRNA amplicon sequencing^40^. Our data supports the idea that at the bacterial family level, metagenome sequencing covered a larger variety of taxonomic groups than 16S rRNA sequencing.

## Conclusions

Overall, the biota of the 2- and 3-year old ash-soil mesocosms resembled that of forest soils more closely than we had expected, even though the total carbon and the total nitrogen concentration (and microbial biomass^10^) was much lower than in a developed forest soil. While bacterial diversity and OTU composition in our mesocosms was very distinct from the forest soils, a wide range of higher level taxonomic groups of prokaryotes and eukaryotes were present. The gene function profile of our ash-soils broadly resembled the B layer of a ‘normal’ temperate forest soil. We had expected to find evidence for a much greater role of chemosynthetic carbon fixation in the ash-soil, but the gene composition and taxonomic composition did not suggest this. However, it is possible that amongst the abundant unclassified prokaryote OTUs in our samples, such activity is actually important.

Bacterial functional gene diversity in the ash-soil mesocosms was also surprisingly similar to that of the forest soils, suggesting that relatively low bacterial taxonomic diversity has little impact on the functional potential of the soil due to redundancy of genes.

Perhaps most importantly in determining the path of development of this system, bryophytes, lichens and cyanobacteria had already begun to establish on the surface of the ash soils by the 24-month stage and were further developed by the 36-month stage^10,26^, and this may be seen as providing the starting point for a rapid positive feedback process in ecosystem development. Also, the relatively moderate pH of the Sakurajima ash may have contributed to the rapid development of the ash system.

It would be interesting to follow the trajectory of developing ash-soil mesocosms over further years to understand how long they take to more closely resemble a developed soil ecosystem. However, it is important to bear in mind that our mesocosm systems are in certain ways limited in simulating natural volcanic ash soil biota succession. The proximity of a developed ecosystem surrounding the mesocosms may mean there was a ready source of dispersal of propagules of microbes and small soil animals, and the spores of bryophytes, which once established are likely a major source of photosynthetic carbon. Nevertheless, studies of volcanic landscape succession have often shown that bryophyte and lichen spores are capable of long distance dispersal on the wind^17–19^. Unlike a ‘real’ volcanic ash field, our mesocosms have also been relatively disadvantaged in terms of arrivals, being cut off by the mesh covering from wind dispersed seeds, insects, and from bird droppings which could bring vascular plants, nutrients, and soil microbes. Additionally, our pots could not readily be colonized from below, whereas buried legacy soils may be important in many natural volcanic ash fields^20^. In ecology, there is an almost inevitable trade-off between control of the experimental conditions, and the complex and confusing realism of a natural ecosystem. Both approaches are important and complimentary, and there is a need for more field based studies—ideally beginning as soon as possible after the eruption itself—to understand the development of volcanic ash into soil ecosystems in a more natural context.

## Materials and methods

### Source of volcanic ash

The volcanic ash used in this study was derived from the Sakurajima volcano, on the southern island Kyushu in Japan (31° 34′ 50″ N 130° 39′ 29″ E). Sakurajima is an andesitic stratovolcano, which in recent decades has been in a continuous state of low level eruption showering ash on its surrounding slopes and for several kilometres beyond^41^. Being andesitic, the ash from Sakurajima is rich in silica and low in calcium and magnesium. Sakurajima ash does not usually contain any large amounts of acids such as sulphuric acid, hydrochloric acid and hydrofluoric acid, although acidic rainfall events around the volcano have sometimes occurred^42^. At time of our sampling, the freshly deposited volcanic ash had a pH of about 5.2, no more acidic than many soils in southern Japan.

### Setting up microcosms, soil sampling, and DNA extraction

After several days of eruption, freshly fallen ash was collected on plastic sheets near the Sakurajima volcano, and sterilized at 200 °C in dry heat in an oven for an hour. We set up several groups of pot mesocosms of this ash at various locations and climates around Japan, in clearings in natural woodland or scrub vegetation (methods described in detail in Kerfahi et al.^10^). Briefly, these locations were (a) the lower slopes of the Sakurajima volcano, is a sparsely wooded area of young pines (*Pinus *sp.) with large openings of bare ash and herbaceous vegetation (mainly *Fallopia *sp.) (b) Takakuma, mixed evergreen broadleaf hill forest some 10 km from Sakurajima, (c) Kamigamo, mixed evergreen broadleaf forest some 600 km away near Kyoto, (d) two sites on Mt Norikura, 800 km northeast of Sakurajima. One site was at 1450 m asl. (above sea level) (referred to as “Norikura_Low” in the manuscript) surrounded by cool climate deciduous broadleaves and conifers (mainly *Larix *sp*.*), the second site was at 2800 m asl. (referred to as “Norikura_High” in the manuscript), surrounded by open dwarf pine (*Pinus pumila*) scrub with many bare areas of loose gravel and scree.

Pots of the sterilized ash were placed in replicate plastic trays (drained by holes, several pots per tray for repeat sampling over several years), the trays placed 5 m apart outside and each tray covered by 1 mm hole size plastic gauze, as described in Kerfahi et al.^10^. Replicates of the pots were sampled from their respective trays at 24 months (2 years) and 36 months (3 years) after being set up. The top 2 cm of the ash in each pot were used for DNA extraction and analysis. To compare functional gene composition of ash soils with natural vegetated soils, we collected one to four cores (same width as the pots), each 5 m apart from one another of the top 2 cm of soil from the adjacent natural ‘forest’ (in some cases more resembling scrub) vegetation, < 200 m from each experimental site. Soil DNA was extracted using the Power Soil DNA extraction kit (MO BIO Laboratories, Carlsbad, CA, USA) and kept at − 20 °C for further processing.

Year 1 samples (12 months) did not yield enough DNA for amplicon or shotgun sequencing. Bacterial community data based on the 16S rRNA sequencing for Year 2 (24 months) are already published as Kerfahi et al.^10^. For a more complete perspective, we added another year’s set of samples for 16S community data, as well as shotgun sequencing the 24 month and 36 month samples for metagenomes. To compare bacterial communities with natural forest soils, we obtained publicly available sequence data from other studies on the same site^10,43^. Details of the samples can be found in Supplementary Table S3 for 16S rRNA data and Supplementary Table S4 for metagenome data.

### Soil physicochemical analysis

To determine total carbon and total nitrogen concentration of samples, an NC analyzer (NC-900; Shimadzu, Kyoto, Japan) was used. pH was measured using pH meters (D-51, Horiba, Kyoto, Japan) after extraction from 10 g of dry ash with 25 mL of ion exchange water^10,26^.

### PCR and sequencing

To amplify 16S rRNA gene from soils, universal bacterial primers 341F (5′-CCTAGGGGNGGCWGCAG-3′) and 805R (5′-GACTACHVGGGTATCTAATCC-3′) were used. Polymerase chain reaction were performed under the following conditions: initial denaturation (95 °C, 3 min) followed by 25 cycles of denaturation (95 °C, 30 s), annealing (55 °C, 30 s), and elongation (72 °C, 30 s), and final extension (72 °C, 5 min). After purifying PCR products using AMPure XP beads (Beckman Coulter, Inc., Brea, CA, USA), index PCR was performed under following conditions: initial denaturation (95 °C, 3 min) followed by 8 cycles of denaturation (95 °C, 30 s), annealing (55 °C, 30 s), and elongation (72 °C, 30 s), and final extension (72 °C, 5 min). PCR products were purified and pooled together and sequenced under Illumina MiSeq platform (Illumina, Inc.). Whole genome shotgun sequencing was carried out by Celemics (Celemics, Inc., Seoul, Korea), using an Illumina NextSeq sequencers (Illumina, Inc.).

### 16S rRNA amplicon sequence processing

Paired end sequences were joined together using pandaseq v.2.8.^44^. Sequences were further processed using Mothur v. 1.42.3.^45^ following the MiSeq SOP (https://www.mothur.org/wiki/MiSeq_SOP). Sequences were aligned and classified based on Silva database v.132.^46^. Since natural forest sample DNAs from Kerfahi et al.^10^ have been amplified using different primer sets from our study and Cho et al.^43^ (Supplementary Table S3), we used only the commonly aligned nucleotide sequences of each read. 16S rRNA sequence reads from this study were deposited in MG-RAST under the project ID of X. Operational taxonomic units (OTUs) were assigned based on 97% similarity. Singleton sequences were removed. The OTU table was subsampled into 10,020 reads per samples for diversity analysis.

### Phylogenetic analysis

Maximum-likelihood tree was generated using FastTree v.2.1.3^47^ based on representative OTU sequences. For evaluation of phylogenetic community assembly processes, standardized effect size measure of the mean nearest taxon distances (SES.MNTD)^48,49^ with 999 randomized null modes were calculated using “ses.mntd” in R package “picante”^50^.

### Whole genome shotgun sequence processing

Metagenome sequences were uploaded to MG-RAST under project IDs of mgp92027, mgp87971, and mgp85837 for further processing. Sequence reads with > 5 low quality base pairs (< 15 phred score) were removed. For taxonomic annotation of sequence reads, the RefSeq database^51^ was used and for functional gene annotation, the SEED database^24^ was used. Sequences were annotated using default settings of MG-RAST (maximum e-value cutoff of 10^–5^, minimum % identity cutoff of 60%, and minimum alignment length cutoff of 15 bp). The Subsystem Function level (the finest level) data was subsampled to 160,910 reads per sample for diversity analysis.

### Statistical analysis

To visualize ecological distance between each sample set, OTU table and level 4 functional gene table were square-root transformed and Bray–Curtis dissimilarities between samples were calculated. nMDS plots were drawn and statistical significance of the effect of sites and time were tested using two way crossed ANOSIM test in PRIMER software v.6 (http://www.primer-e.com) with 999 permutations^52^. To compare the Bray–Curtis dissimilarity between the 2nd-year ash soils and forest soils in comparison with the Bray–Curtis dissimilarity between the 3rd-year ash soils and forest soils, we performed t test. P values were adjusted using the Benjamini and Hochberg’s method^53^. To compare soil physicochemical conditions, relative abundance of bacterial taxa, relative abundance of functional genes, and bacterial taxonomic and functional gene diversity between the ash soil and natural forest, ANOVA test was performed. Where the results are significant, Tukey’s HSD test was performed as a posthoc test. For soil physicochemical factors, conditions for ANOVA tests were in some cases not formally met, but given the very clear distinct differences in C and N (ash soils consistently have only 1/10 to 1/100 as much C and N as the forest soils) we present the results here to provide further evidence for the patterns shown.

## Supplementary Information

Supplementary Information S2.

Supplementary Information S1.

Supplementary Information S3.

## Data Availability

The datasets generated during the current study are available in the metagenomics RAST (rapid annotation using subsystems technology) server under the project numbers mgp92027, mgp87971, and mgp85837. For further information on MG-RAST sample accession numbers, refer to Supplementary Table S3 and S4. Authors can confirm that all relevant data are included in the article and/or its supplementary information files.
