# Supplementary material for: Metagenomic analysis reveals rapid development of soil biota on fresh volcanic ash
Source: Sci Rep. 2020 Dec 8;10:21419. doi: 10.1038/s41598-020-78413-z (PMC7723037; doi:10.1038/s41598-020-78413-z)
Supplement: Supplementary file 1 — Supplementary Information S2. [file 41598_2020_78413_MOESM1_ESM.docx]

**Metagenomic analysis reveals rapid development of soil biota on fresh volcanic ash**

Hokyung Song^1^, Dorsaf Kerfahi^2^, Koichi Takahashi^3^, Sophie L Nixon^1^, Binu Mani Tripathi^4^, Hyoki Kim^5^, Ryunosuke Tateno^6^*, Jonathan Adams^7^*

1. Department of Earth and Environmental Sciences, the University of Manchester, Manchester, United Kingdom
2. School of Natural Sciences, Department of Biological Sciences, Keimyung University, Daegu, 42601, Republic of Korea
3. Department of Biology, Faculty of Science, Shinshu University, Matsumoto, 390-8621, Japan
4. Korea Polar Research Institute, Incheon, Republic of Korea
5. Celemics Inc., 19F, Bldg. A, BYC High city, 131, Gasandigital 1-ro, Gwumcheon-gu, Seoul, 153-718, Korea
6. Field Science Education and Research Center, Kyoto University, Kyoto, 606-8502, Japan
7. School of Geography and Ocean Sciences, Nanjing University, Nanjing, 210023, Jiangsu Province, China

* co-corresponding authors

**Supplementary Information**

**Supplementary figures**





**Supplementary Fig. S1**. Boxplots of total carbon concentration (%), total nitrogen concentration (%), and pH. “Second” corresponds to the second year ash soil samples. “Third” corresponds to the third year ash soil samples.

 **Supplementary Fig. S2**. The Bray-Curtis dissimilarity between the 2^nd^-year ash soils and forest soils in comparison with the Bray-Curtis dissimilarity between the 3^rd^-year ash soils and forest soils. Bray-Curtis dissimilarity was calculated based on square-root transformed OTU composition. T-test results are shown above each bar. At Kamigamo, the difference was significant, but not at Norikura and Sakurajima.


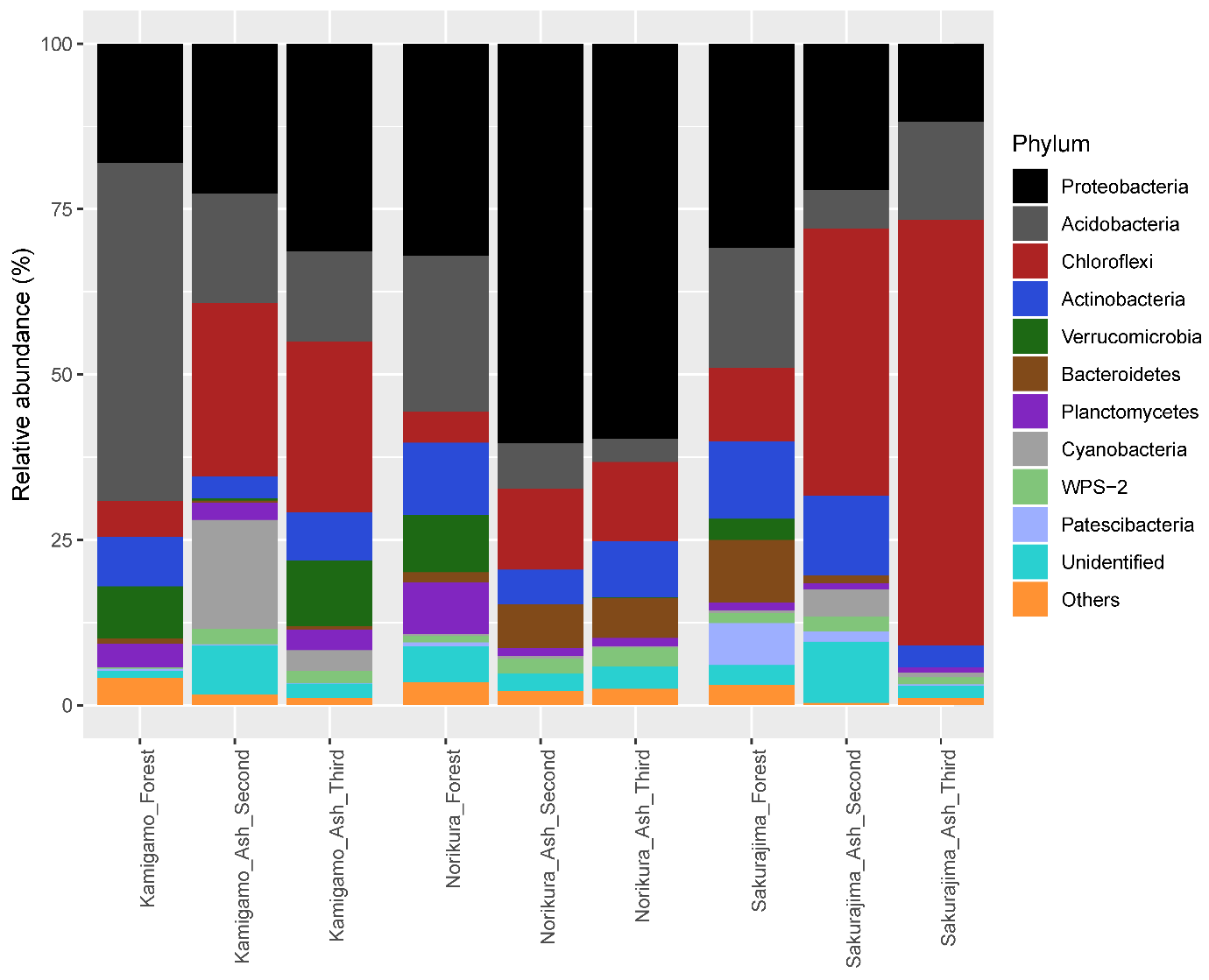


**Supplementary Fig. S3**. Averaged relative abundance of the bacterial phyla based on 16S rRNA gene amplicon sequencing. “Unidentified” includes those that were unclassified, unidentified or with no cultured representatives at the class level. “Others” includes taxa that were only present in a minor proportion.


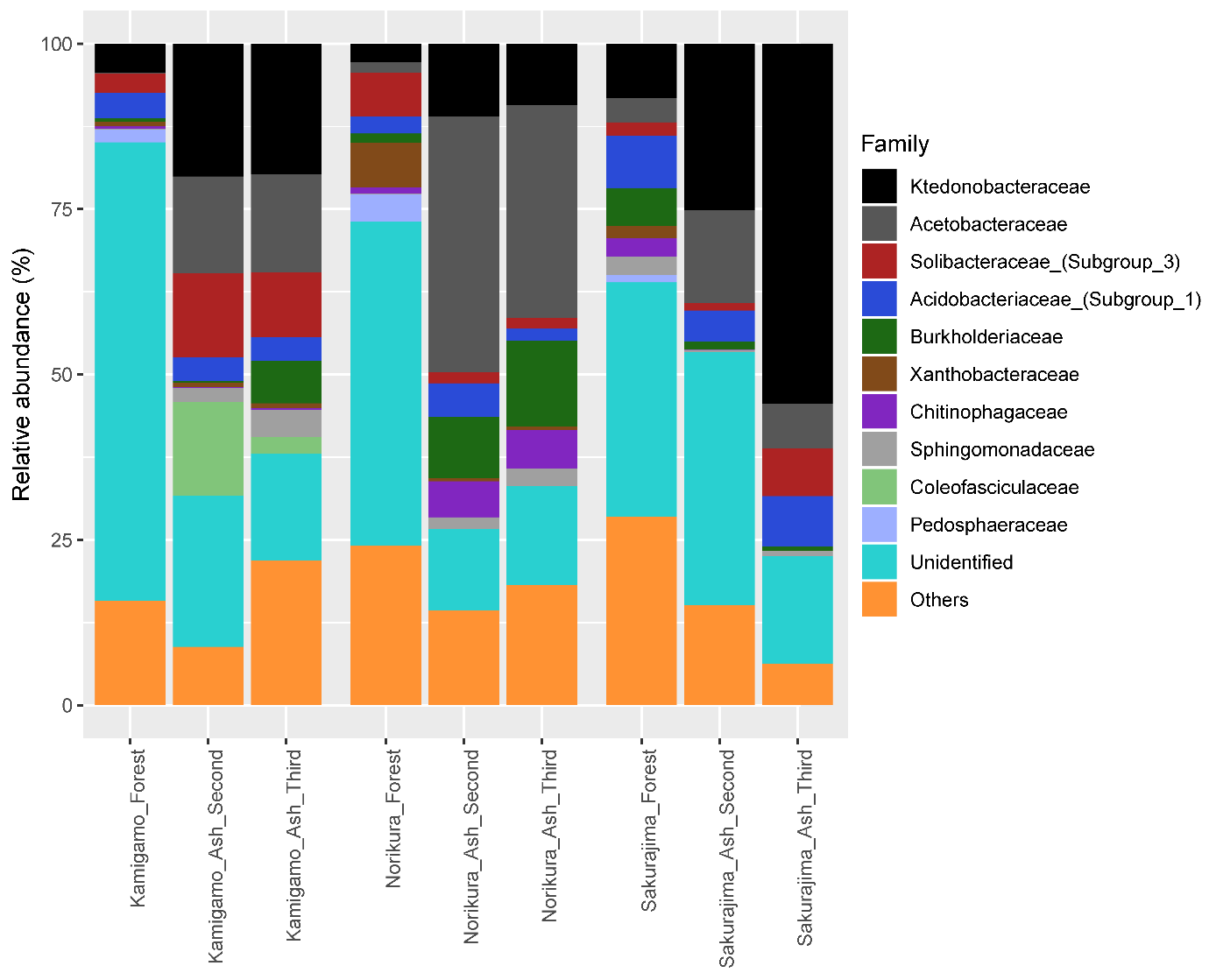


**Supplementary Fig. S4.** Averaged relative abundance of bacterial families based on 16S rRNA amplicon sequencing. “Unidentified” includes those that were unclassified, unidentified or with no cultured representatives at the family level. “Others” includes taxa that were only present in a minor proportion.


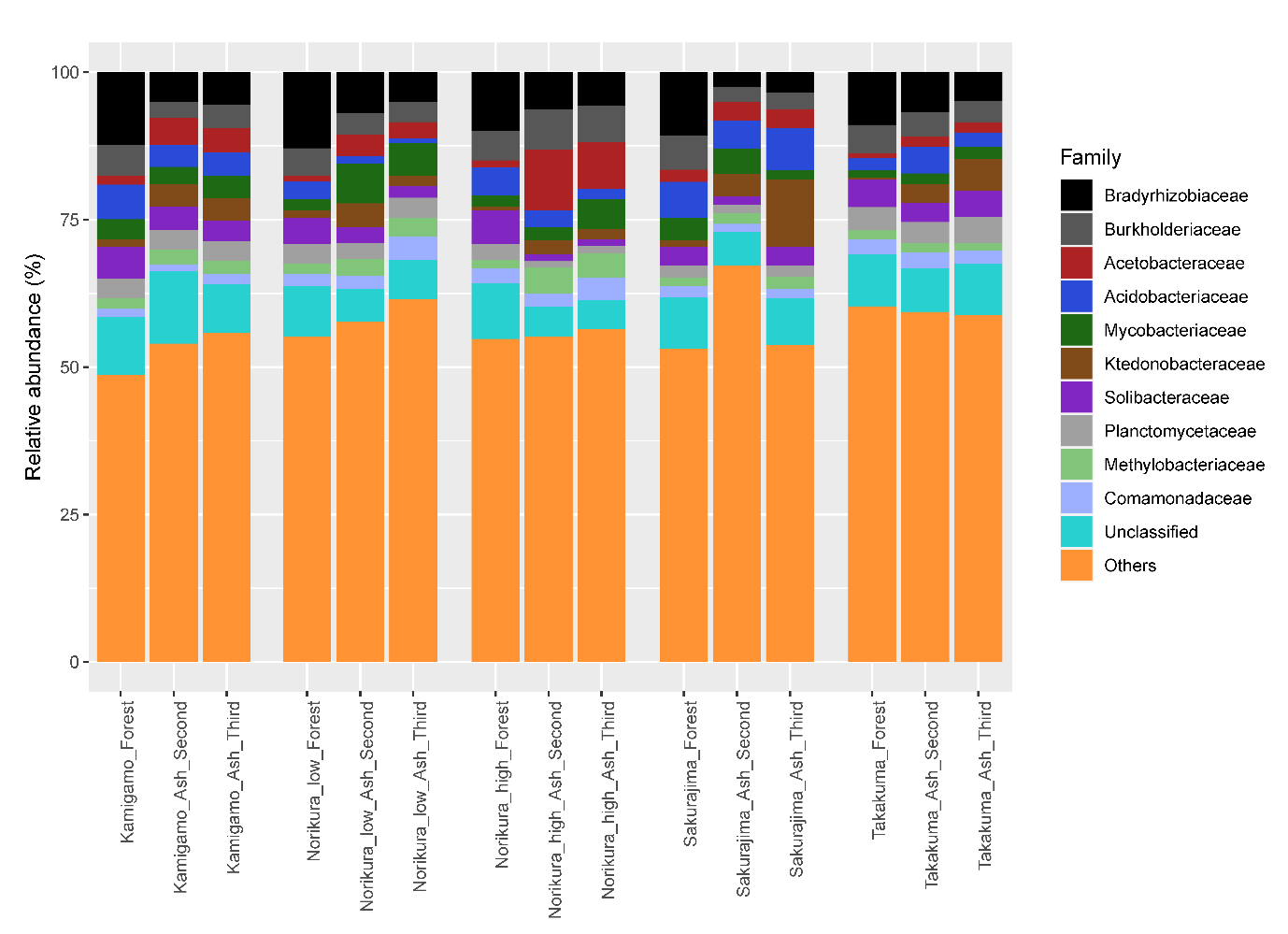


**Supplementary Fig. S5.** Averaged relative abundance of bacterial families based on metagenome sequencing.


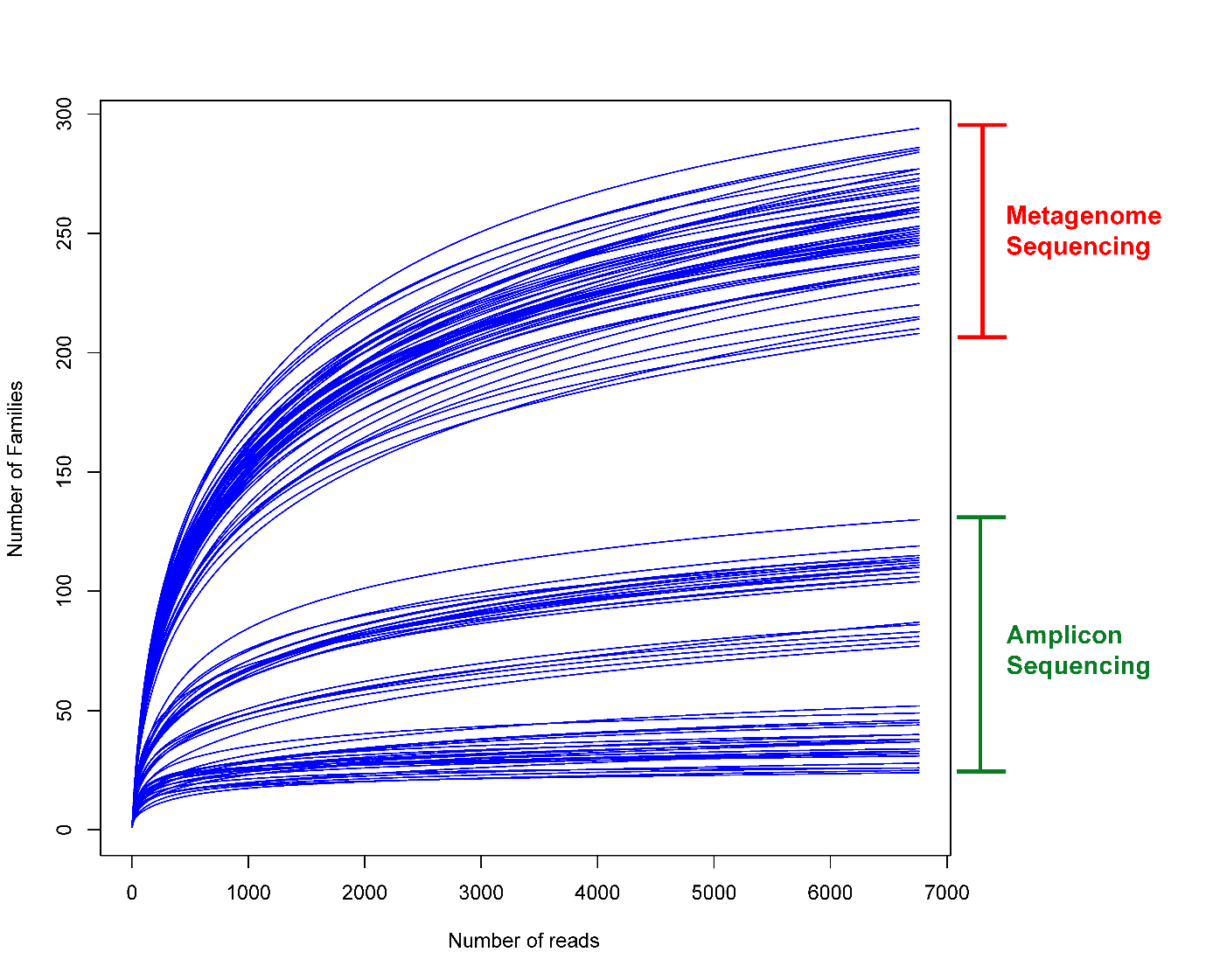


**Supplementary Fig. S6.** Family diversity accumulation curve. Samples were rarefied with 6,760 reads per sample. Sequences that were unclassified, unidentified or with no cultured representatives at the family level were removed prior to rarefaction.

**

**

**Supplementary Fig. S7.** SES.MNTD value in the ash soil and forest soil in the study sites.





**Supplementary Fig. S8.** The number of eukaryotic phyla assigned based on metagenome data. Sequences were subsampled to 597,667 reads per sample before counting the number of phyla.


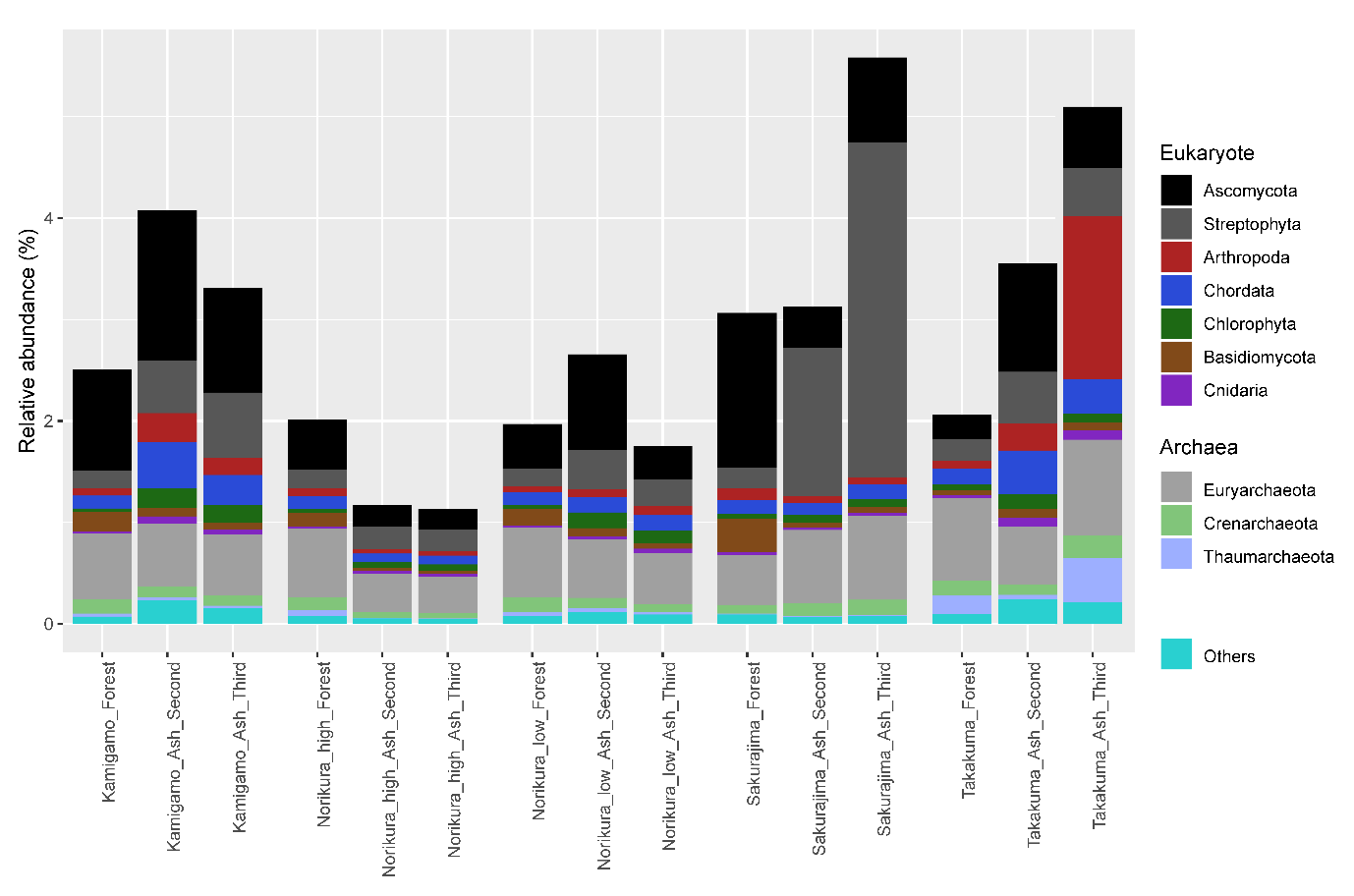


**Supplementary Fig. S9.** Averaged relative abundance of archaeal and eukaryotic metagenome sequences, assigned by phyla (%). Note that not all gene phylum assignments may be accurate due to limitations in the NCBI database assignments of complex genomes (e.g. Cnidaria, Chordata).





**Supplementary Fig. S10.** The Bray-Curtis dissimilarity between the 2^nd^-year ash soils and forest soils in comparison with the Bray-Curtis dissimilarity between the 3^rd^-year ash soils and forest soils. Bray-Curtis dissimilarity was calculated based on the Subsystem level4 functional genes. T-test results are shown above each bar.

**Supplementary tables**

**Supplementary Table S1**. Two way crossed ANOSIM test results of distances between samples calculated based on bacterial OTU composition

| **Test for differences between time** | | |
| --- | --- | --- |
| Global R: 0.809 | p = 0.001 |  |
| Pairwise tests | | |
|  | R | p |
| 2^nd^ year ash soil – natural forest | 1 | 0.001 |
| 3^rd^ year ash soil – natural forest | 1 | 0.001 |
| 2^nd^ year ash soil – 3^rd^ year ash soil | 0.698 | 0.001 |
|  | | |
| **Test for differences between sites** | | |
| Global R: 1 | p = 0.001 |  |
| Pairwise tests |  |  |
|  | R | p |
| Sakurajima – Takakuma | 1 | 0.001 |
| Sakurajima – Kamigamo | 1 | 0.001 |
| Sakurajima – Norikura | 1 | 0.001 |
| Takakuma – Kamigamo | 1 | 0.001 |
| Takakuma – Norikura | 1 | 0.001 |
| Kamigamo – Norikura | 1 | 0.001 |

**Supplementary Table S2**. Two way crossed ANOSIM test results of distance between samples calculated based on the Subsystem level4 functional gene composition

| **Test for differences between time** | | |
| --- | --- | --- |
| Global R: 0.872 | p = 0.001 |  |
| Pairwise tests | | |
|  | R | p |
| 2^nd^ year ash soil – natural forest | 1 | 0.001 |
| 3^rd^ year ash soil – natural forest | 1 | 0.001 |
| 2^nd^ year ash soil – 3^rd^ year ash soil | 0.702 | 0.001 |
|  | | |
| **Test for differences between sites** | | |
| Global R: 0.999 | p = 0.001 |  |
| Pairwise tests |  |  |
|  | R | p |
| Sakurajima-Kamigamo | 0.962 | 0.001 |
| Sakurajima-Norikura_low | 0.972 | 0.001 |
| Sakurajima-Norikura_high | 0.953 | 0.002 |
| Sakurajima-Takakuma | 1 | 0.001 |
| Kamigamo-Norikura_low | 0.895 | 0.001 |
| Kamigamo-Norikura_high | 0.958 | 0.002 |
| Kamigamo-Takakuma | 0.924 | 0.001 |
| Norikura_low-Norikura_high | 0.949 | 0.001 |
| Norikura_low-Takakuma | 0.985 | 0.001 |
| Norikura_high-Takakuma | 1 | 0.001 |

**Supplementary Table S3 and S4 are provided separately as Excel formatted files.**
